# Crime Films

**Published:** 1920-10-30

**Authors:** 


### Crime Films.

The recent references in these columns to the psychological dangers to children of cinema exhibitions are emphasised by the report, since issued, of the British Board of Film Censors, of which Mr. T. P. O'Connor is President. We alluded particularly to the evil effects of films, which include murder, suicide, and violence, and the Board of Censors state that one of the most difficult questions is that of crime films, which make a strong appeal to the imagination of the public. The Board considers these films threaten to become a danger to the reputation of the cinema. The examiners found themselves flooded with films, in some cases running to twenty episodes, in which inhuman monsters using all kinds of mysterious methods of assassination were to be shown week by week over a long period. The Board found the evil assuming such proportions that it was decided that no serial dealing with crime should be examined except as a whole, that no serial in which crime was the dominant feature and not merely an episode in the story would be passed by the Censor, and that no film should be passed in which the methods of crime were set forth and formed the chief theme. This rule is to be applied even in cases where at the end of the film retribution is supposed to have fallen on the criminal, and equally when the detective element is subordinate to the criminal interest, or when actual crime is treated from the comic point of view. Stories dealing with "costume" crime, however, such as cowboy films and Mexican robberies, are placed in a different category and regarded simply as dramatic and thrilling adventures.

In endeavouring to check indecorum in dress no figure is passed in which the dress appears to be meant to be indecent or suggestive. With regard to films dealing with venereal disease and the white slave traffic, the Board has decided to withhold its certificate from all such subjects, even when they have been dealt with on the stage or are matters of public discussion at the moment.

As regards censorship generally, the report states that the tendency is for the subjects to become of increasing difficulty and complexity. The Board is guided by the main broad principles that nothing should be passed which is calculated to demoralise an audience; that can teach methods of or extenuate crime; that can undermine the teachings of morality; that tend to bring the institution of marriage into contempt or lower the sacredness of family ties.

The difficulties of the Board are thus plainly demonstrated in this report, and it is clear that much must be passed as fit for adult audiences which is by no means suitable for children. It is not possible for official authorities to overcome this, and the only hope is that parents and guardians of children will sooner or later learn to realise the dangers of such unchildlike excitements upon young and sensitive minds. There is only one thing beyond reasonable censorship such as we believe is being exercised by the Board, that could usefully be done, and that is definitely to prohibit attendance of children up to a certain age beyond a certain hour of the evening. The law quite rightly safeguards children regarding hours of labour, why should it not safeguard them regarding hours of so-called entertainment.

